# Effect of fish scale-derived biofiller loading on the mechanical, microstructural and moisture-resistance behavior of snake plant fiber/epoxy composites

**DOI:** 10.1039/d6ra05489g

**Published:** 2026-08-11

**Authors:** Barshan Dev, Md Ashikur Rahman, Apurbo Das, Md Azmol Hossain Emon, Sakib Iqbal Akand, Abhi Karmakar, Md Zillur Rahman

**Affiliations:** a Department of Textile Engineering, BGMEA University of Fashion and Technology Dhaka 1230 Bangladesh barshan.butex@gmail.com; b Department of Apparel Manufacturing and Technology, BGMEA University of Fashion and Technology Dhaka 1230 Bangladesh; c Department of Industrial and Production Engineering, Dhaka University of Engineering and Technology Gazipur 1707 Bangladesh; d Department of Mechanical Engineering, Ahsanullah University of Science and Technology Dhaka 1208 Bangladesh md.zillur.rahman.phd@gmail.com

## Abstract

Sustainable composites based on natural fibers and bio-derived fillers offer a promising alternative to conventional synthetic materials. This study systematically investigates the influence of fish scale (FS) bio-filler incorporation on the mechanical performance, microstructural characteristics, and moisture-uptake behavior of snake plant (SP) fiber-reinforced epoxy composites. Composites were fabricated using the hand lay-up technique with a constant SP fiber content (20 wt%) and varying FS filler loadings (0–20 wt%). Mechanical properties, including tensile, flexural, impact, and hardness behavior, as well as fracture morphology, chemical interactions, and water-absorption kinetics and apparent Fickian diffusion behavior, were evaluated. Results showed that the filler-free composite exhibited superior overall mechanical performance, achieving the highest tensile strength (87.66 MPa) and modulus (4.78 GPa), flexural strength (114.28 MPa) and modulus (6.45 GPa), and impact strength (20.12 kJ m^−2^), attributed to effective fiber–matrix interaction and matrix continuity. Increasing FS content caused a gradual decline in tensile, flexural, and impact properties, particularly at higher filler loadings, due to weakened interfacial adhesion and increased defect formation. However, hardness improved with FS incorporation, reaching a maximum value of 72.54 Shore D at 10 wt% FS. SEM analysis revealed a transition from rough, well-bonded fracture surfaces to brittle, crack-dominated morphologies with increasing filler content, while FTIR indicated physical interactions without new chemical bonds. At 120 h, water absorption decreased by 36%, from 17.50% in S1 to 11.20% in S5. However, apparent diffusion coefficients showed no consistent trend, indicating reduced moisture retention but not necessarily slower transport. Overall, 5–10 wt% FS provided the best performance balance, although long-term moisture durability requires further validation. These findings support the development of lightweight, sustainable composites for non-load-bearing and semi-structural applications.

## Abstract 2

Fish-scale biofiller reduced 120 h water uptake by 36% and increased hardness at 10 wt% loading, but excess filler lowered tensile and impact properties. An optimum 5–10 wt% loading balanced mechanical performance and moisture resistance.

## Graphical abstract

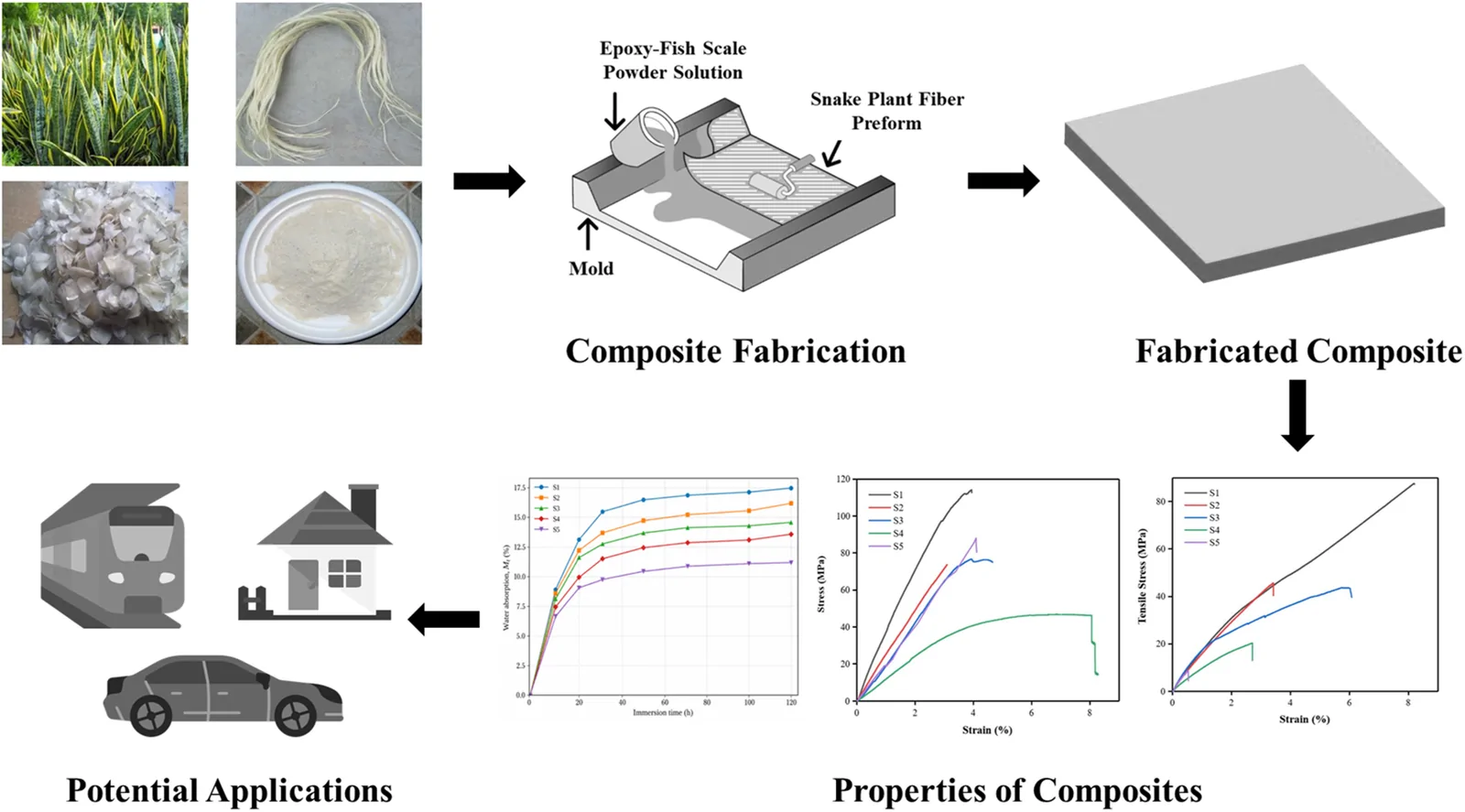

## 1 Introduction

Natural fiber-reinforced polymer composites have attracted considerable attention in recent years due to their sustainability, low density, biodegradability, cost-effectiveness, and acceptable mechanical performance for semi-structural and non-structural applications.^1–3^ The increasing environmental concerns associated with petroleum-based materials and synthetic reinforcements have encouraged researchers to explore lignocellulosic fibers and biomass-derived fillers as viable alternatives.^4–6^ Natural fibers such as jute, sisal, kenaf, bamboo, pineapple leaf fiber, and snake plant fiber exhibit favorable specific strengths and stiffnesses, making them promising reinforcements in polymer matrices.^7,8^ However, despite their advantages, challenges such as hydrophilicity, poor interfacial adhesion, and property variability still limit their broader application. To address these issues, hybridization and the incorporation of functional bio-fillers have emerged as effective strategies to enhance the overall performance of natural fiber composites.^2,6^

Bio-fillers derived from animal sources, agricultural waste, and plant-based residues have been increasingly utilized to improve mechanical, thermal, and physical properties while maintaining environmental compatibility.^9,10^ These fillers not only reduce material costs but also support waste valorization and circular economy initiatives. Previous studies have consistently demonstrated that incorporating bio-fillers can improve the performance of natural fiber-reinforced composites; however, the extent of improvement strongly depends on the filler type, particle characteristics, loading level, and compatibility with the polymer matrix. For instance, Salmah *et al.*^11^ developed polylactic acid (PLA) biocomposites incorporating both treated and untreated coconut shell powder (CSP) and evaluated their tensile behavior. The results showed that acrylic acid-treated PLA/CSP composites exhibited enhanced tensile strength and modulus, along with a decrease in elongation at break compared to their untreated counterparts. Rahman *et al.*^9^ fabricated jute–glass fiber-reinforced polyester composites with 5–20 wt% fish scale powder and found that 10 wt% filler provided the best balance of mechanical, thermal, and environmental performance, demonstrating its potential for sustainable applications. Angdembe *et al.*^12^ investigated hemp fiber-reinforced epoxy composites with walnut shell filler and reported that the inclusion of filler significantly improved creep resistance, fatigue life, dielectric properties, and wear performance compared to the filler-free composite. Nagaprasad *et al.*^13^ examined the effect of tamarind seed and date seed fillers (TSF/DSF) on the mechanical performance of vinyl ester composites across a wide range of filler loadings (0–50 wt%). Their findings indicated that composites containing 10 wt% TSF/DSF exhibited the best tensile, flexural, and impact properties among the compositions.

Moreover, Rosamah *et al.*^14^ investigated the mechanical performance of polyester composites reinforced with oil palm shell (OSP) at filler contents of 1, 2, and 3 wt%. Their results showed that the composite containing 3 wt% OSP exhibited enhanced tensile and flexural properties compared to the lower filler loadings. Kowshik *et al.*^15^ investigated the mechanical behavior of glass fiber-reinforced epoxy composites incorporating eggshell (ES) fillers in uncarbonized, carbonized, and hybridized forms at 10 wt% loading. Their findings indicated that composites containing carbonized FS fillers exhibited comparatively superior tensile and flexural properties. Dinesh *et al.*^16^ fabricated and characterized jute fiber-reinforced epoxy composites incorporating wood dust fillers. They reported that the uniform dispersion of fine padauk wood dust particles improved matrix adhesion and enhanced mechanical properties, while the presence of coarser rosewood dust particles increased the thermal stability of the composites. Karthik *et al.*^17^ studied calabash (*Crescentia cujete*) fiber-reinforced epoxy composites with natural fillers and found improved mechanical strength, reduced thermal conductivity, good fiber–matrix adhesion, moderate crystallinity, and notable antibacterial activity, indicating strong potential for sustainable structural and biomedical applications. Surface treatment of fibers and fillers has further been shown to significantly enhance composite performance. Chemical treatments such as alkali, silane, and acetylation reduce hydrophilicity and improve fiber–matrix adhesion.^18–21^ Studies have demonstrated that treated natural fibers exhibit better interfacial bonding, resulting in improved tensile, flexural, and impact properties. When combined with bio-fillers, treated fiber composites often show synergistic effects, leading to enhanced load transfer efficiency and reduced interfacial defects.^20,21^

Despite these advances, the current literature reveals three important research gaps. First, most published studies have focused on either plant-derived bio-fillers or synthetic filler systems. In contrast, animal-derived bio-fillers, particularly fish scale waste, remain comparatively underexplored in natural fiber-reinforced epoxy composites. Second, snake plant fiber has received limited attention as a reinforcement in hybrid bio-filled composite systems despite its favorable mechanical characteristics. Third, there is a lack of systematic investigations evaluating how progressively increasing fish scale bio-filler content affects the coupled mechanical, microstructural, and moisture-transport behavior of snake plant fiber/epoxy composites under identical processing conditions. In particular, the distinction between reduced gravimetric water uptake and changes in apparent diffusion rate has not been adequately addressed. Consequently, the relationships among filler loading, matrix continuity, interfacial defects, short-term moisture retention, and overall property balance remain insufficiently understood.

Therefore, the present study addresses these gaps by investigating SP/epoxy composites with a constant fiber fraction (20 wt%) and progressively increasing FS filler content from 5 wt% to 20 wt%. Mechanical properties, including tensile, flexural, impact, and hardness, are evaluated alongside fracture morphology, chemical interactions, and water-absorption behavior to clarify the structure–property relationships. Water-uptake kinetics are further analyzed using a comparative plane-sheet Fickian model to estimate operational, geometry-dependent apparent diffusion coefficients and distinguish gravimetric moisture retention from normalized moisture transport. By integrating natural fiber reinforcement with fish-scale biofiller, this study aims to identify filler-loading ranges that balance mechanical integrity, surface hardness, and reduced short-term water uptake for sustainable non-load-bearing and semi-structural applications.

## 2 Materials and methods

### 2.1 Materials

**Fig. 1:**
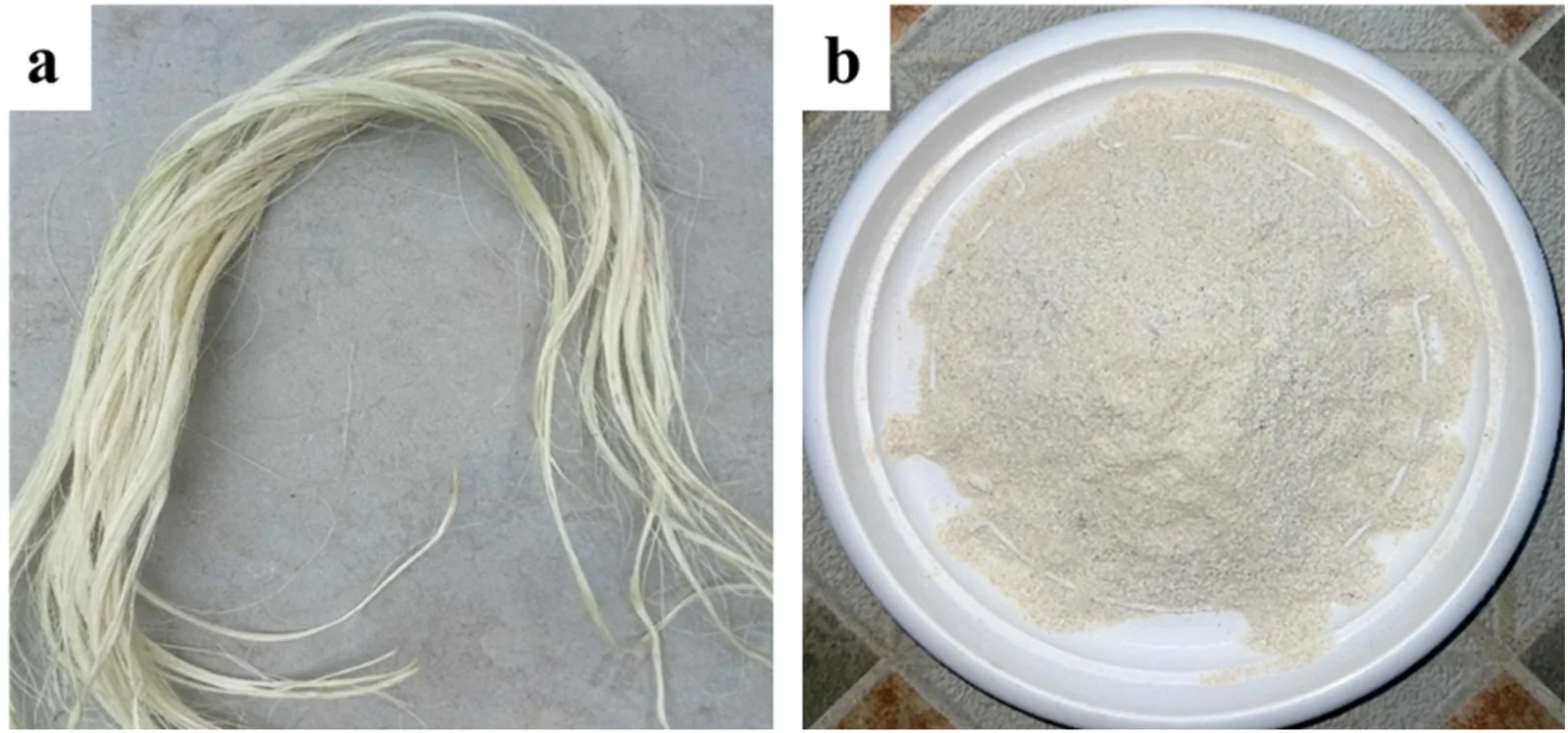
(a) Extracted SP fibers and (b) fish scale powder.

Snake plants (*Sansevieria trifasciata*) were sourced locally from Gazipur, Bangladesh (Fig. 1). The plants were first cut into suitable lengths to aid fiber extraction. The SP leaves were immersed in water for 3 days for retting. After retting, the fibers were manually extracted and rinsed three times thoroughly with distilled water. Finally, the cleaned fibers were oven-dried at 70 °C for 3 hours (Fig. 1a). Fish scale (FS) powder was used as fillers in this study. The FS was collected from a local market in Gazipur, Bangladesh. Then, the FS was cleaned with distilled water to eliminate any odor and pollutants. Subsequently, the cleaned FS was sun-dried for 6 h, then oven-dried at 100 °C for 3 h. After this, the FS was crushed into a fine powder using an electric grinder (Fig. 1b). The epoxy resin (Araldite LY556) was used as the matrix with a density of 1.1 g cm^−3^ and a viscosity of 12 000–13 000 cP, while the hardener (Aradur HY951) was used as a catalyst to accelerate chemical reactions and as a curing agent.

### 2.2 Development of preform

The extracted SP fibers were stored in a dry, controlled-atmosphere condition at 25 (±2) °C and 65 (±2)% relative humidity for 3 h before use in this study. Following this, elementary fibers were separated from technical fibers using a hand-combining device. Then, the fibers were cut to a desired length (175 mm) to manufacture the UD preform measuring 175 mm × 110 mm. SP fibers were aligned in a single direction (*i.e.*, UD) within a wooden frame and securely clamped at both ends to maintain the UD orientation of the fiber layer. The fibers were then slightly moistened and subjected to hot pressing at 20 bar, 120 °C, and 10 minutes. Five UD preforms were made with the same SP fiber weight percentage (20 wt%) (Table 1).

**Table 1:** Weight percentages of SP fiber, FS filler, and epoxy resin

Composite	SP fiber (wt%)	FS filler (wt%)	Epoxy-hardener (wt%)
S1	20	0	80
S2	20	5	75
S3	20	10	70
S4	20	15	65
S5	20	20	60

### 2.3 Fabrication of composites

**Fig. 2:**
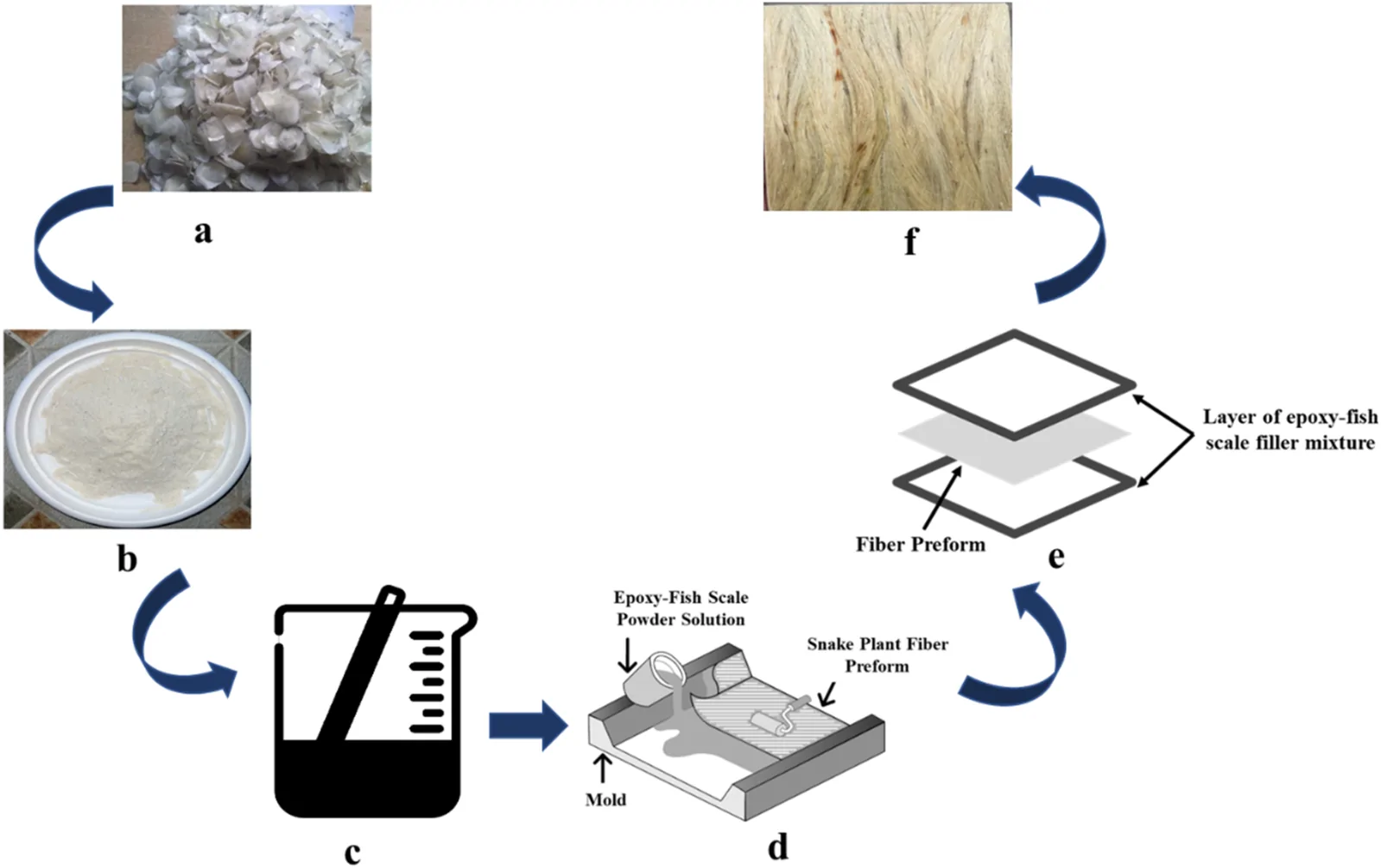
Fabrication of composites: (a) fish scale, (b) fish scale powder, (c) mixing of epoxy and fish scale powder, (d) composite fabrication by hand lay-up, (e) layer of fiber preform and mixture, and (f) fabricated composite laminate.

The composites in this study were fabricated using the manual hand lay-up technique (Fig. 2). A mold cavity measuring 175 mm × 110 mm × 3 mm was used to stack the composite laminate, with plastic plates placed on both sides of the laminate to prevent it from sticking. All composites were fabricated following the same manufacturing conditions with a single lamination. The epoxy resin was prepared with a resin-to-hardener ratio of 3 : 1. Four different weight percentages of FS fillers, 5, 10, 15, and 20% by weight, were added to the epoxy matrix in the formulations as a matrix substitution. The composition was manually mixed for five minutes using a glass rod stirrer in a beaker to achieve a homogeneous mixture of the epoxy resin, hardener, and FS fillers. Then, half of the mixture was applied evenly to the mold plate, and the SP fiber preform was placed over the epoxy–FS powder layer. After placing the fiber preform, the remaining mixture was applied evenly over the preform. A hand roller was used to ensure the preform layer was homogeneous. In addition, the roller eliminated any trapped air bubbles in the composite laminate. The composite arrangement was then covered with another plate, and a 245 N load was applied for 24 h at room temperature. Subsequently, the load was released, and the composite laminate was removed from the mold. The composites were labeled according to the FS filler weight percentage. Table 2 represents the formulations of FS fillers in the epoxy matrix for manufacturing SP fiber composites.

### 2.4 Tensile test

Tensile tests were conducted using a universal testing machine (AG-X plus, Japan) equipped with a 20 kN load cell, following ASTM D638-03. Specimens measuring 165 mm in length, 20 mm in width, and 3 mm in thickness were prepared as per the standard. The cross-head speed of the machine remained constant at 5 mm min^−1^ throughout the test. The average value and standard error were reported in this study for all mechanical tests (tensile, flexural, impact, and hardness) based on at least 5 test specimens.

### 2.5 Flexural test

Three-point bending tests were conducted on composite specimens measuring 125 mm × 20 mm × 3 mm using a universal testing machine (AG-X plus, Japan) equipped with a 10 kN load cell, following ASTM D790. The bending support length (load span) of 50 mm and the crosshead speed of 1.4 mm min^−1^ were maintained as per the standard.

### 2.6 Impact test

A Charpy impact test was conducted in accordance with ASTM D256. Specimens with V-notches measuring 64 mm × 13 mm × 3 mm were obtained from manufactured composite laminates for this testing. A pendulum weighing 2.634 kg was released to strike the sample at an angle of 150°, and an indicator was used to precisely determine the point of contact between the pendulum and the sample. Subsequently, the impact energy was calculated using the chart provided with the impact tester.

### 2.7 Hardness test

The hardness test was conducted on composite specimens measuring 35 mm × 15 mm × 3 mm using a durometer hardness tester (Shore D), in accordance with ASTM D2240. The test used a hardened steel rod indenter with a 1.25 mm diameter and a 30° conical tip with a 0.1 mm tip radius. During testing, an approximate load of 45 N was applied.

### 2.8 Fourier transform infrared spectroscopy (FTIR)

FTIR was used to identify the functional groups present in FS fillers, SP fibers, epoxy resin, and the composites. The powdered samples were mixed with pre-dried KBr and compressed into pellets under identical preparation conditions. A blank KBr pellet was used for background correction, while baseline correction and spectral normalization were performed using the instrument software. The instrument was calibrated according to the manufacturer's recommended procedure. Spectra were recorded using an FTIR spectrophotometer (L1600400 Spectrum Two FT-IR/DTGS, PerkinElmer, UK) over 400–4000 cm^−1^, with 34 scans per sample at a resolution of 4 cm^−1^. The spectra were interpreted mainly by comparing peak positions and functional-group characteristics.

### 2.9 Scanning electron microscope (SEM)

The fracture surfaces of specimens subjected to tensile testing were analyzed using the scanning electron microscope (SEM, SU1510, Hitachi, Japan). Cross-sections were utilized to accurately depict the internal microstructure of the specimens. The specimen pieces were mounted on brass bits or aluminum stubs. All specimens were then coated with a thin layer of gold particles for approximately 20 minutes using an AGAR sputter coater to enhance electron conductivity. After this, the SEM micrographs of the specimens were captured.

### 2.10 Water absorption and diffusion analysis

Water absorption was measured using a modified procedure based on ASTM D570-98. Rectangular specimens measuring 64 mm × 12.7 mm × 3 mm were oven-dried at 65 °C and weighed to determine the initial dry mass, *W*_0_. The specimens were then immersed in distilled water at the specified test temperature. At immersion times of 10, 20, 30, 50, 70, 100, and 120 h, each specimen was removed, surface-dried consistently with a lint-free absorbent cloth, weighed immediately to determine *W*_*t*_, and returned immediately to the distilled water bath. Gravimetric water absorption at time *t*, *M*_*t*_, was calculated using eqn (1).1
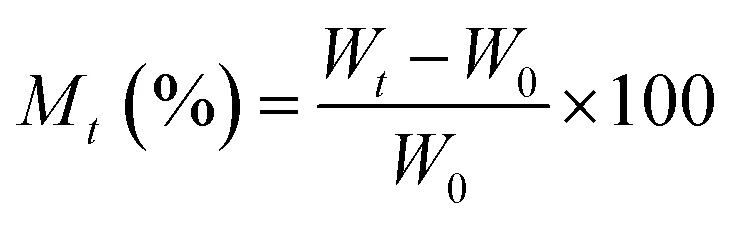
where *W*_0_ is the initial dry mass and *W*_*t*_ is the mass after immersion for time *t*.

Although the specimens were not edge-sealed and contained heterogeneous reinforcement phases, a one-dimensional plane-sheet Fickian diffusion model was employed as a comparative approach to evaluate the relative moisture transport behavior among different formulations. The moisture uptake kinetics were analyzed using the analytical solution of Fick's second law for a plane sheet exposed on both broad surfaces, originally derived by Crank^22^ and subsequently applied to fiber-reinforced composites by Hassanpour and Karbhari:^23^2
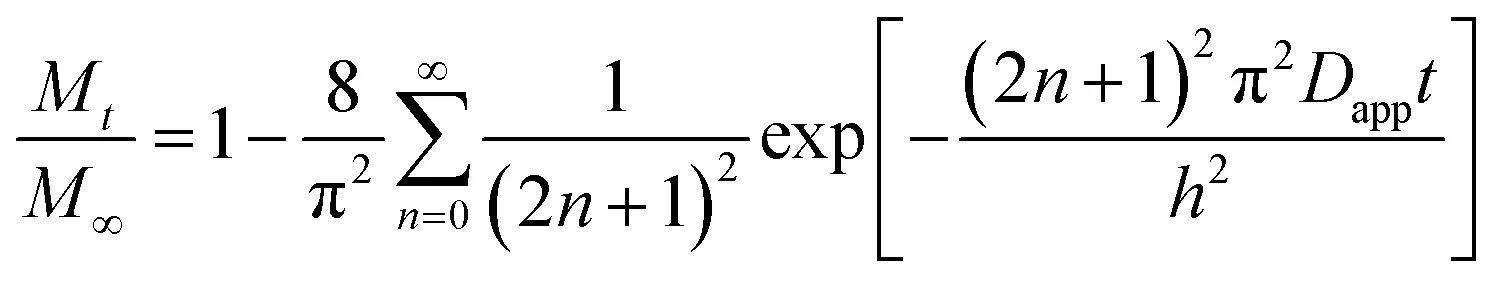
where *M*_*t*_ is the moisture uptake at time *t*, *M*_∞_ is the equilibrium moisture uptake predicted by the model, *D*_app_ is the apparent Fickian diffusion coefficient, *t* is the immersion time in seconds, and *h* is the specimen thickness in meters.

Since equilibrium saturation was not reached within the experimental duration, *M*_120_ was used only as a practical normalization reference for comparative fitting among formulations. Therefore, the *D*_app_ was obtained through nonlinear least-squares fitting of eqn (2) to all experimental data points. The quality of the fitting was assessed using the coefficient of determination (*R*^2^) and root-mean-square error (RMSE). The short-time linear approximation was not applied because the first recorded measurement at 10 h already corresponded to approximately 0.51–0.60 of *M*_120_, providing insufficient early-stage resolution for reliable estimation of the initial diffusion slope. Furthermore, due to the unsealed specimen edges and unidirectional laminate architecture, moisture ingress occurred through multiple pathways, resulting in an anisotropic transport response. Therefore, the calculated *D*_app_ values should be considered operational, geometry-dependent parameters suitable for comparative analysis rather than intrinsic directional diffusivities. Accurate determination of directional diffusion coefficients would require edge-sealed specimens, different specimen orientations/geometries, or a comprehensive three-dimensional inverse modeling approach.^23,24^

## 3 Results and discussion

### 3.1 Tensile properties

**Fig. 3:**
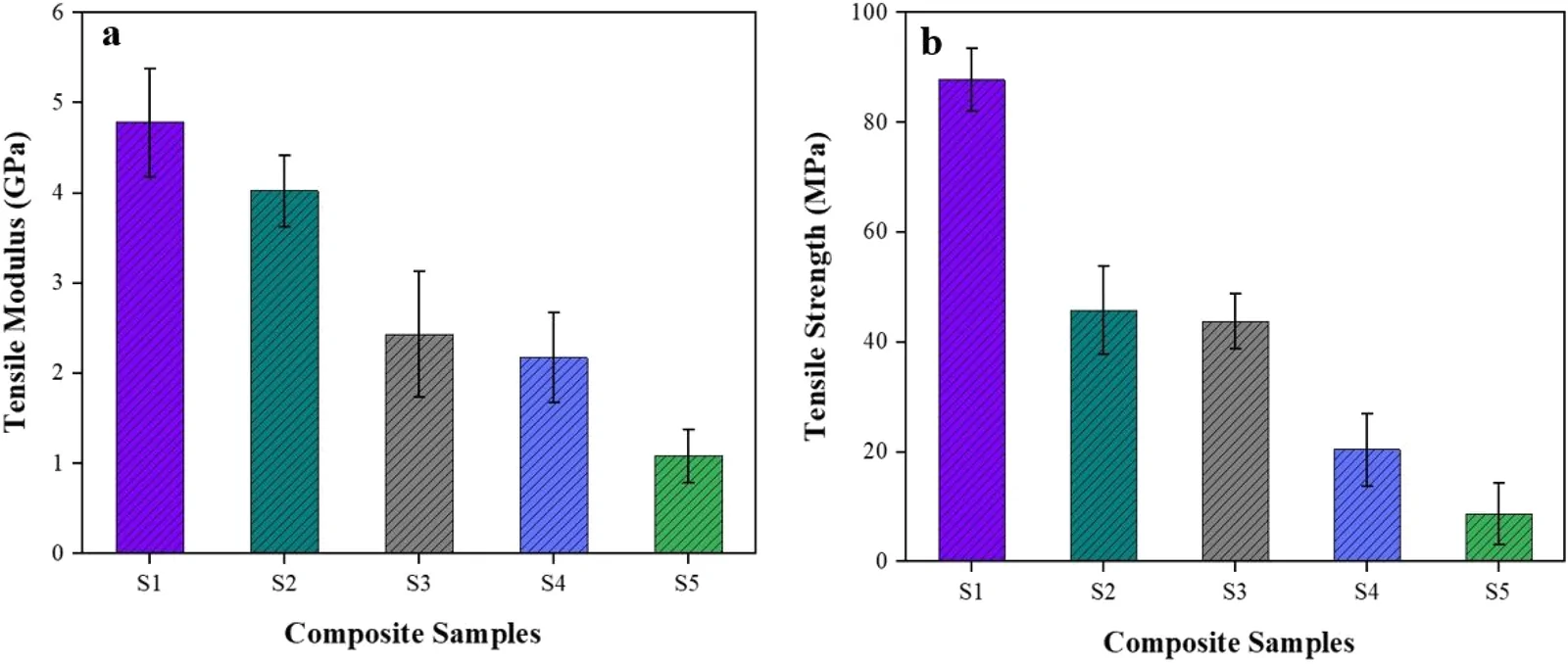
Tensile properties of fabricated SP/epoxy composites with varying contents of FS bio-filler: (a) tensile modulus and (b) tensile strength.

The tensile properties of snake plant fiber-reinforced epoxy composites filled with varying fish scale bio-filler contents are depicted in Fig. 3. The tensile behavior shows a clear and progressive deterioration with increasing filler loading. The filler-free SP/epoxy composite (S1) exhibits the maximum tensile modulus (4.78 GPa) and maximum tensile strength (87.66 MPa), indicating effective stress transfer between the SP fibers and the epoxy matrix. Upon incorporation of 5 wt% FS bio-filler (S2), the tensile modulus decreased to 4.02 GPa, while the tensile strength dropped sharply to 45.70 MPa, representing an almost 48% reduction compared to S1. Further increasing FS content to 10 wt% (S3) reduced the modulus to 2.43 GPa and strength to 43.70 MPa. At 15 wt% FS (S4), the modulus and strength declined further to 2.17 GPa and 20.32 MPa, respectively. The maximum filler loading of 20 wt% (S5) results in a substantial decrease in tensile modulus to 1.08 GPa and tensile strength to 8.65 MPa. These results clearly demonstrate that both tensile modulus and tensile strength decrease significantly with increasing FS filler content, even from the initial 5 wt% addition.

The superior tensile properties of S1 can be attributed to the optimized balance between fiber reinforcement and matrix continuity. With 20 wt% SP fiber and 80 wt% epoxy, the matrix fraction is sufficient to ensure proper fiber wetting, strong interfacial adhesion, and minimal void formation.^9,25^ Effective stress transfer from the relatively ductile epoxy matrix to the stiffer SP fibers enables higher stiffness (4.78 GPa) and strength (87.66 MPa). However, the introduction of FS bio-filler reduces the epoxy content while adding a rigid, discontinuous particulate phase to the matrix.^9,26^ Fish-scale bio-filler, primarily composed of hydroxyapatite and collagen-based constituents, may not exhibit strong interfacial compatibility with the epoxy unless surface-treated.^27,28^ Consequently, the filler particles can act as stress concentration sites. In S2, despite the relatively low 5 wt% filler loading, the reduction in matrix content (75 wt%) combined with possible particle–matrix interfacial debonding significantly lowers tensile strength to 45.70 MPa and modulus to 4.02 GPa. This indicates that the negative effect of stress concentration and weakened fiber–matrix interaction outweighs any potential stiffening effect of the rigid bio-filler.^29^

With further increase in FS content from 10 wt% (S3) to 20 wt% (S5), the deterioration in tensile performance becomes more pronounced due to multiple interacting mechanisms. First, the progressive reduction in epoxy content (70 wt% in S3, 65 wt% in S4, and 60 wt% in S5) limits the matrix's ability to effectively encapsulate both SP fibers and FS particles. Insufficient matrix coverage increases the likelihood of void formation and poor interfacial bonding.^27,28^ Second, higher filler loading promotes particle agglomeration, disrupting matrix continuity and creating defect clusters that facilitate crack initiation and propagation under tensile loading. Third, excessive particulate inclusion interferes with efficient stress transfer from the matrix to the SP fibers, thereby reducing the reinforcing efficiency of the fiber phase.^28,30^ As a result, tensile modulus decreases progressively from 2.43 GPa (S3) to 2.17 GPa (S4) and finally to 1.08 GPa (S5), while tensile strength drops sharply from 43.70 MPa to 20.32 MPa and ultimately to 8.65 MPa. The drastic reduction at 20 wt% FS suggests that the composite microstructure becomes filler-dominated rather than fiber-reinforced, leading to premature failure and severely compromised mechanical integrity.^9^ Overall, the tensile results confirm that increasing the FS bio-filler content adversely affects stiffness and strength due to reduced matrix continuity, weakened interfacial bonding, filler agglomeration, and enhanced stress concentration.

#### 3.1.1 Tensile stress–strain curves

**Fig. 4:**
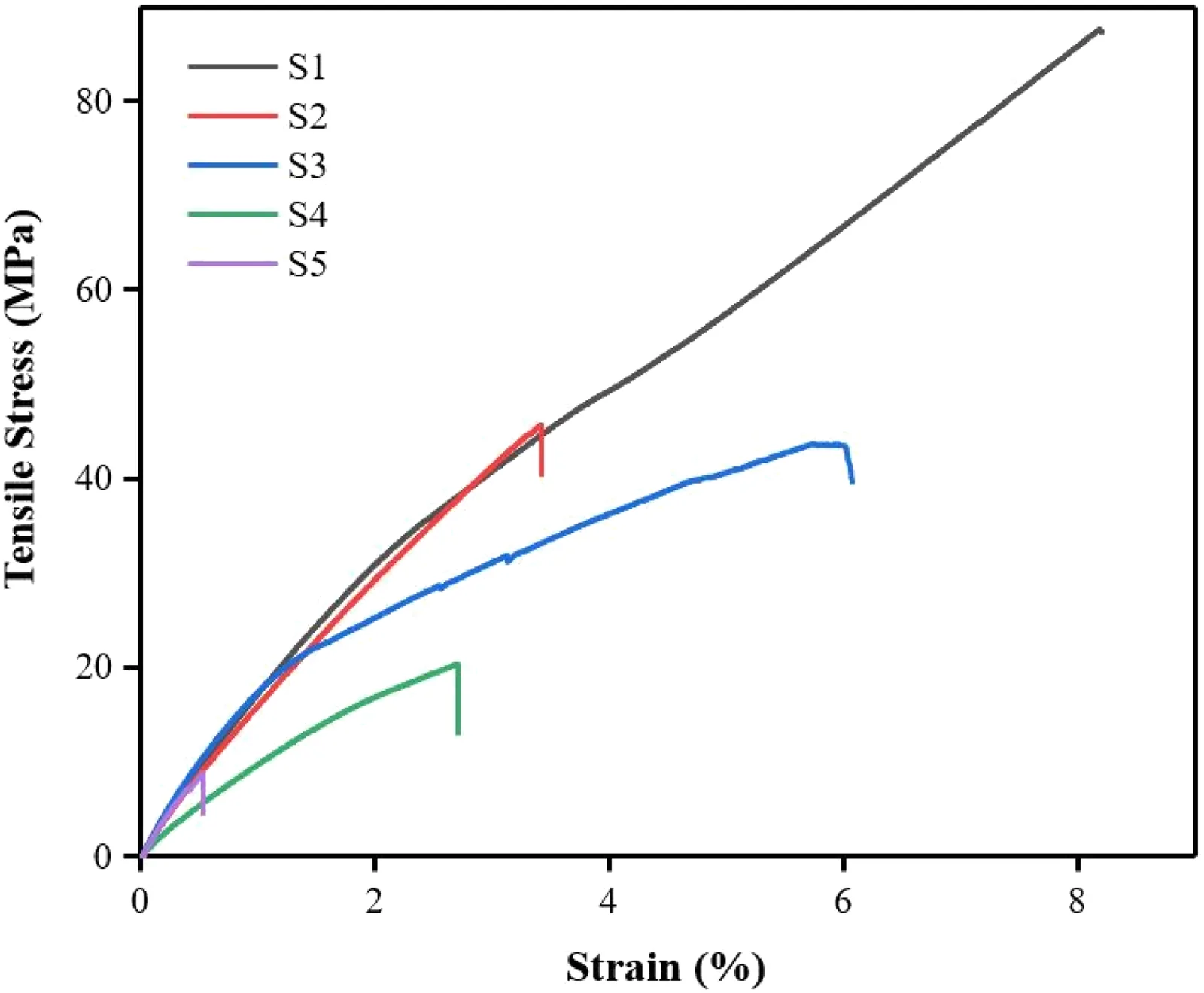
Tensile stress–strain curves of fabricated composites with varying FS bio-fillers.

The tensile stress–strain curves shown in Fig. 4 demonstrate a clear dependence of mechanical performance on the FS bio-filler content in SP fiber-reinforced epoxy composites. The filler-free composite exhibits the highest tensile strength (87.66 MPa) and the highest strain at break (8–8.2%), indicating superior load-bearing capacity and relatively ductile behavior. This can be attributed to effective stress transfer between the SP fibers and the epoxy matrix, supported by good interfacial adhesion and sufficient matrix continuity,^27^ as explained in Section 3.1. With the incorporation of 5 wt% FS (S2), the tensile strength drops significantly to 45.70 MPa, and the strain at break decreases to ∼3.3–3.5%, reflecting reduced ductility and stiffness. A similar trend is observed for S3 (10 wt% FS), where the tensile strength slightly decreases to 43.70 MPa, while the strain at break increases moderately to ∼6.0–6.2%, suggesting some retention of deformability despite reduced stiffness.

As the FS content increases further, the deterioration in tensile behavior becomes more pronounced. The S4 composite (15 wt% FS) shows a substantial reduction in tensile strength (20.32 MPa) and strain (∼2.5–2.8%), indicating limited load-bearing capability and early failure. The highest filler loading (S5, 20 wt% FS) exhibits the poorest performance, with tensile strength dropping to 8.65 MPa and strain at break reduced to only ∼0.6–0.8%, reflecting a highly brittle nature. This result indicates that increasing FS bio-filler content adversely affects both tensile strength and elongation. This degradation is mainly due to reduced epoxy matrix continuity, poor interfacial bonding between filler and matrix, increased stress concentration, and possible filler agglomeration, all of which hinder efficient stress transfer and lead to premature failure of the composites,^25,31^ as described in Section 3.1.

### 3.2 Flexural properties

**Fig. 5:**
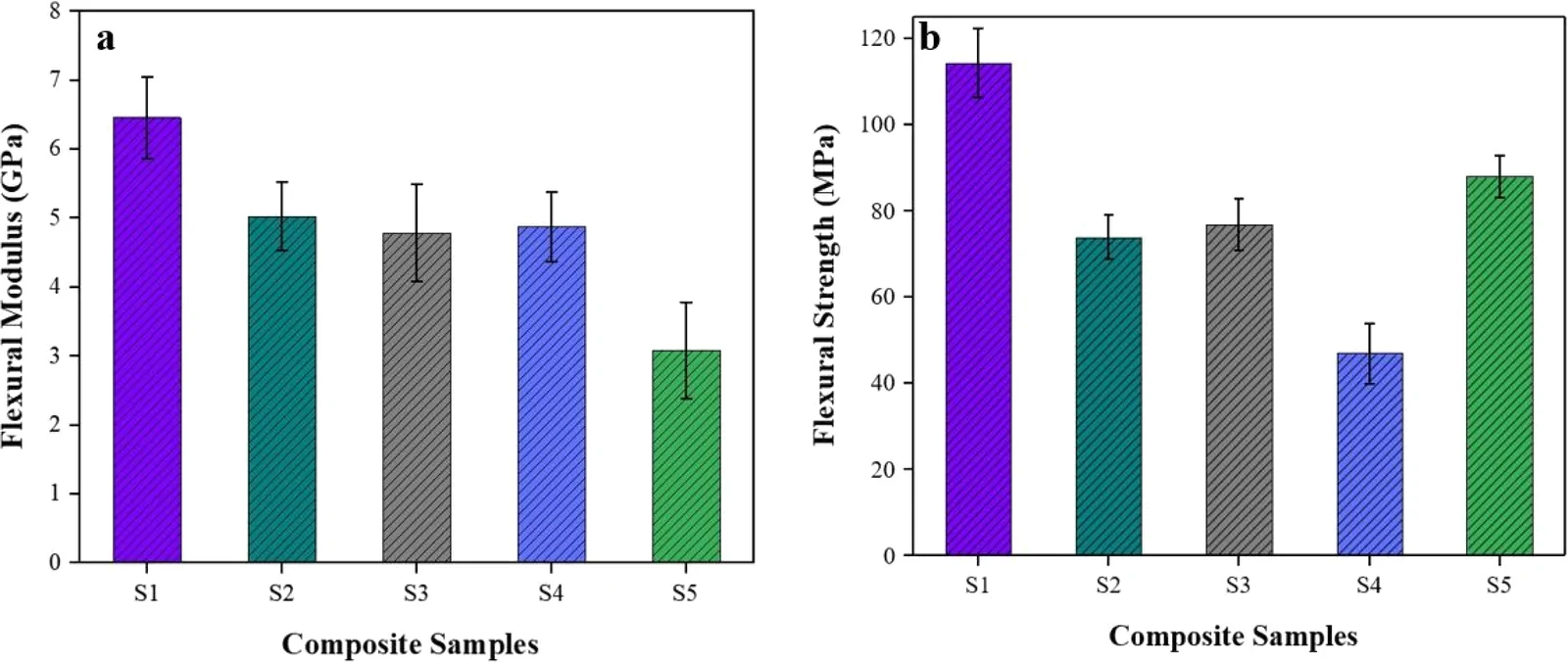
Flexural properties of fabricated SP/epoxy composites with varying contents of FS bio-fillers: (a) flexural modulus and (b) flexural strength.

The flexural properties of SP/epoxy composites filled with different FS contents are illustrated in Fig. 5. The flexural modulus decreases with increasing filler loading, consistent with the trend observed in the tensile modulus. However, the flexural strength does not follow the same pattern as the tensile strength and shows a non-consistent variation with filler content. This indicates that while stiffness reduction due to filler incorporation is consistent across both loading conditions, the flexural strength is influenced by additional factors, such as stress distribution, filler dispersion, and interfacial characteristics. The filler-free composite (S1) exhibits the highest flexural modulus of 6.45 GPa and flexural strength of 114.28 MPa, demonstrating efficient stress transfer between SP fibers and the epoxy matrix under bending conditions. With the incorporation of 5 wt% FS bio-filler (S2), the flexural modulus decreases to 5.02 GPa, while the flexural strength drops significantly to approximately 73–75 MPa. At 10 wt% filler loading (S3), the modulus further decreases to 4.78 GPa; however, the flexural strength shows a slight increase compared to S2, reaching approximately 76–78 MPa. This minor improvement suggests a marginal contribution of the rigid filler phase to load-bearing capacity despite reduced matrix continuity.^9,32^ At 15 wt% FS (S4), the flexural modulus shows a slight recovery to 4.87 GPa, whereas the flexural strength decreases considerably to about 46–50 MPa, indicating deterioration in structural integrity. At the highest filler loading of 20 wt% (S5), the modulus drops significantly to 3.08 GPa, while the flexural strength increases again to approximately 88–92 MPa. This contrasting behavior suggests that although stiffness is compromised by reduced matrix continuity, partial stress-bearing from the increased filler content may improve strength relative to S4.^33–35^

The superior flexural performance of S1 can be attributed to the optimized interactions between SP fibers and the continuous epoxy matrix. Under bending, the composite experiences both tensile and compressive stresses, requiring strong interfacial bonding and uniform matrix distribution. With 80 wt% epoxy, S1 ensures effective wetting and encapsulation of SP fibers, minimizing voids and enabling efficient stress transfer, resulting in maximum modulus and strength. Upon introducing FS bio-filler, the epoxy content decreases progressively (75 wt% in S2, 70 wt% in S3, 65 wt% in S4, and 60 wt% in S5), disrupting matrix continuity. The FS bio-filler, primarily composed of hydroxyapatite and collagen-based constituents, may exhibit limited compatibility with the epoxy matrix without surface treatment.^27^ This leads to particle–matrix debonding and localized stress concentrations under flexural loading. The initial reduction in strength at S2 indicates that these negative effects outweigh the filler's reinforcing effect. At higher filler loadings, additional microstructural factors contribute to property degradation. Reduced matrix content limits proper encapsulation of both fibers and filler particles, increasing void formation and stress heterogeneity.^28,32^ Moreover, higher filler content promotes agglomeration, particularly at 15 wt% (S4), where clustering acts as critical defect sites for crack initiation and propagation, resulting in the lowest flexural strength. Although S5 shows improved strength compared to S4, the significant reduction in modulus indicates that the composite becomes increasingly filler-dominated, compromising stiffness and effective fiber reinforcement.^32,36^

The flexural behavior indicates that FS bio-filler incorporation adversely affects bending performance at filler loadings above low levels. The reduction in matrix continuity, increased filler agglomeration, weakened interfacial bonding, and inefficient stress transfer collectively contribute to the deterioration of flexural properties. These findings partially align with the tensile results, confirming that uncontrolled incorporation of FS bio-filler is detrimental to the mechanical integrity of SP/epoxy composites.

#### 3.2.1 Flexural stress–strain curves

**Fig. 6:**
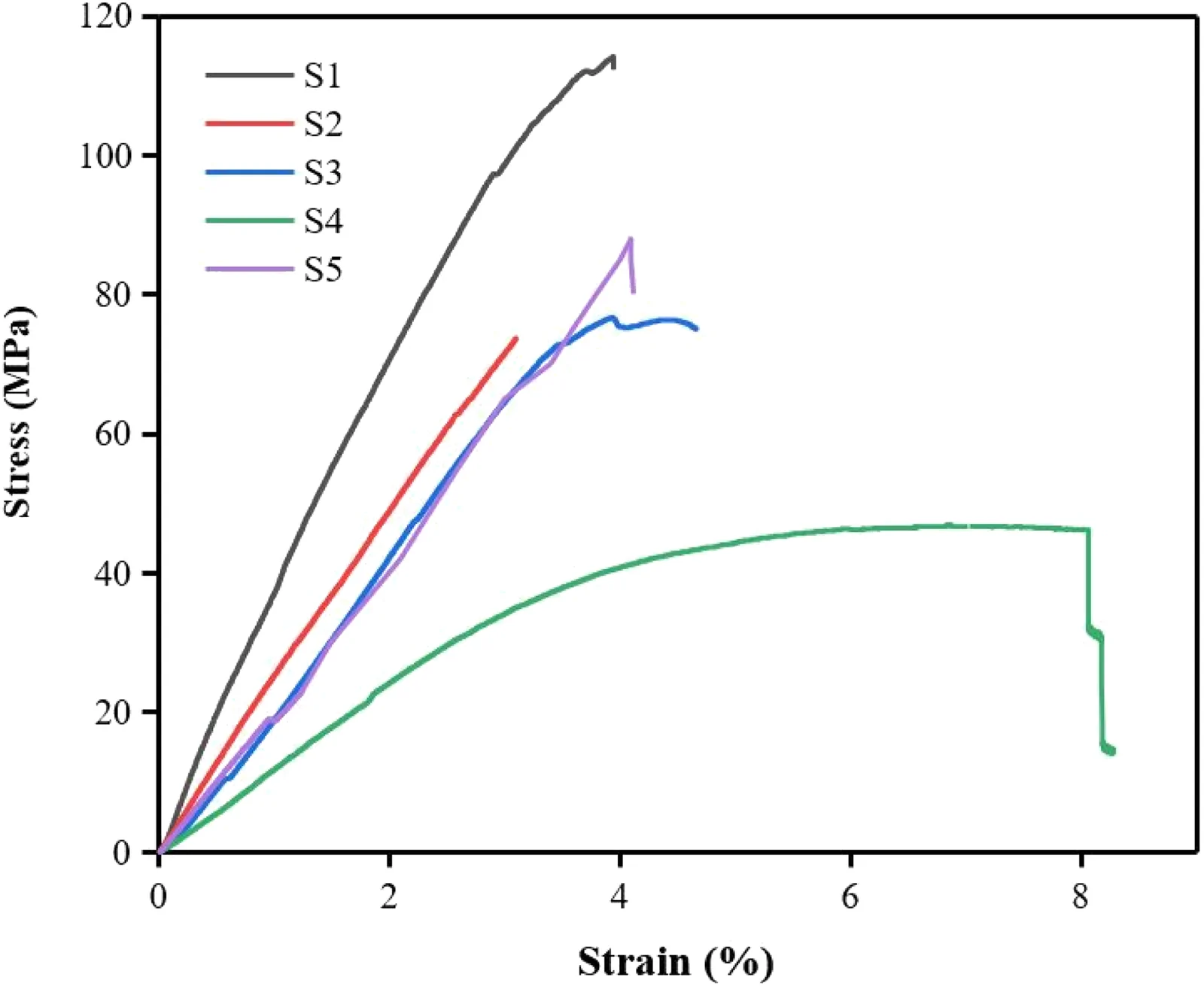
Flexural stress–strain curves of manufactured composites.

The flexural stress–strain curves presented in Fig. 6 demonstrate the influence of FS bio-filler content on the bending behavior of SP fiber-reinforced epoxy composites. The filler-free composite (S1) exhibits the highest flexural strength of 114.28 MPa, along with a strain at break of approximately ∼3.8–4.0%, indicating superior stiffness and load-bearing capacity under flexural loading. This behavior is attributed to efficient stress transfer between SP fibers and the continuous epoxy matrix, supported by strong interfacial adhesion.^33–35^ With the incorporation of 5 wt% FS bio-filler (S2), the flexural strength decreases significantly to approximately 73.85 MPa, while the strain at break reduces to around ∼2.8–3.0%, indicating reduced bending stiffness and earlier failure. At 10 wt% FS (S3), the flexural strength shows a slight improvement to approximately 76.8 MPa, with a corresponding increase in strain at break to about ∼4.2–4.5%, suggesting a marginal contribution of the filler to load-bearing capacity despite reduced matrix continuity.^31^ At 15 wt% FS (S4), the flexural strength drops significantly to approximately 43.83 MPa, while the strain increases significantly to about ∼7.5–8.2%, indicating reduced structural integrity but increased deformability under bending. In contrast, at the highest filler loading of 20 wt% (S5), the flexural strength increases to ∼87.85 MPa again, with a strain at break of 4.0–4.2%, suggesting a partial stress-bearing contribution from the increased filler content. The results reveal a non-consistent variation in flexural strength with increasing FS bio-filler content, while strain behavior varies with filler dispersion and matrix continuity.^9,26^

### 3.3 Impact strength

**Fig. 7:**
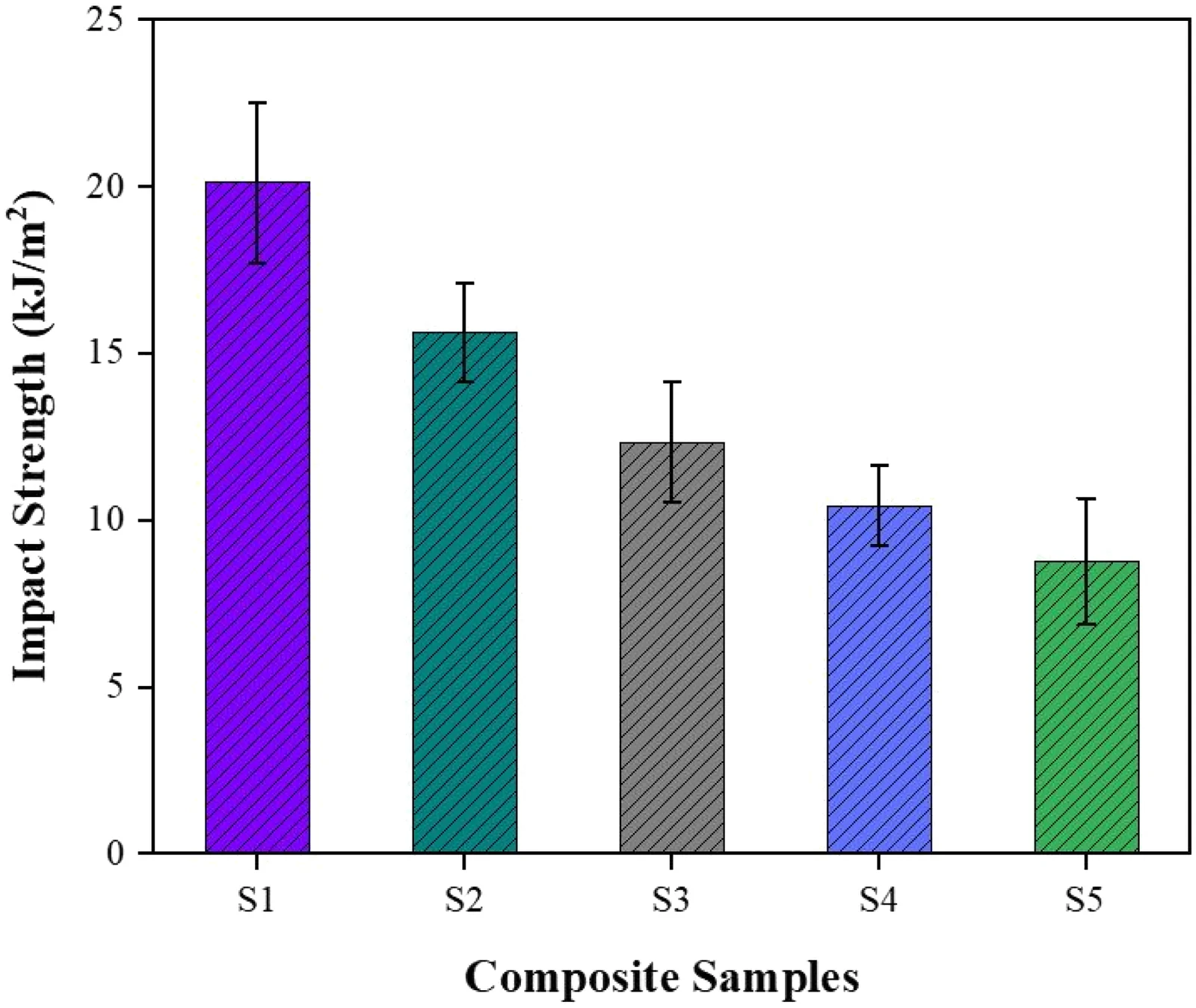
Impact strength of fabricated SP/epoxy composites with varying contents of FS bio-filler.

The impact strength of composites with varying FS filler contents is illustrated in Fig. 7. The impact strength of composites decreases gradually with increasing FS filler content. The composite S1 demonstrates the maximum impact strength of 20.12 kJ m^−2^, confirming that the optimized balance between SP fiber reinforcement and a continuous epoxy matrix provides effective resistance against crack initiation and propagation. Upon incorporation of 5 wt% FS bio-filler in S2, the impact strength decreases to 15.62 kJ m^−2^. Further increasing the filler loading to 10 wt% in S3 reduces the value to 12.32 kJ m^−2^, followed by 10.43 kJ m^−2^ at 15 wt% FS in S4, and ultimately 8.76 kJ m^−2^ at the maximum filler loading of 20 wt% in S5. The reduction from 20.12 kJ m^−2^ (S1) to 8.76 kJ m^−2^ (S5) represents a substantial loss in toughness, clearly indicating that increasing FS bio-filler content adversely affects the composite's ability to absorb impact energy. This consistent trend suggests that even the initial addition of FS filler significantly compromises impact resistance.^9,32^

The maximum impact strength for S1 indicates an optimized fiber–matrix architecture where the continuous epoxy phase effectively wets and binds the snake plant fibers, enabling efficient stress redistribution under sudden loading. In this configuration, energy is dissipated through mechanisms such as matrix deformation, fiber pull-out, fiber fracture, and crack deflection, which collectively delay catastrophic failure.^33,34^ However, the incorporation of FS bio-filler progressively reduces the impact resistance. This continuous decline is primarily attributed to the reduction in epoxy content (from 80 wt% in S1 to 60 wt% in S5), which limits matrix continuity and its capacity for plastic deformation under impact. The rigid, mineral-rich FS particles introduce internal heterogeneities within the polymer matrix and may create weak interfacial regions in the absence of strong chemical compatibility, thereby acting as stress-concentration sites.^34,37^ At higher filler loadings, particle agglomeration and insufficient matrix encapsulation further promote microvoid formation and rapid crack propagation, shifting the failure mode toward brittle fracture.^9^ Consequently, increasing FS content transforms the composite from a relatively tough, fiber-reinforced system to a more brittle, filler-influenced structure, resulting in the substantial reduction in impact strength from S1 to S5.

### 3.4 Hardness

**Fig. 8:**
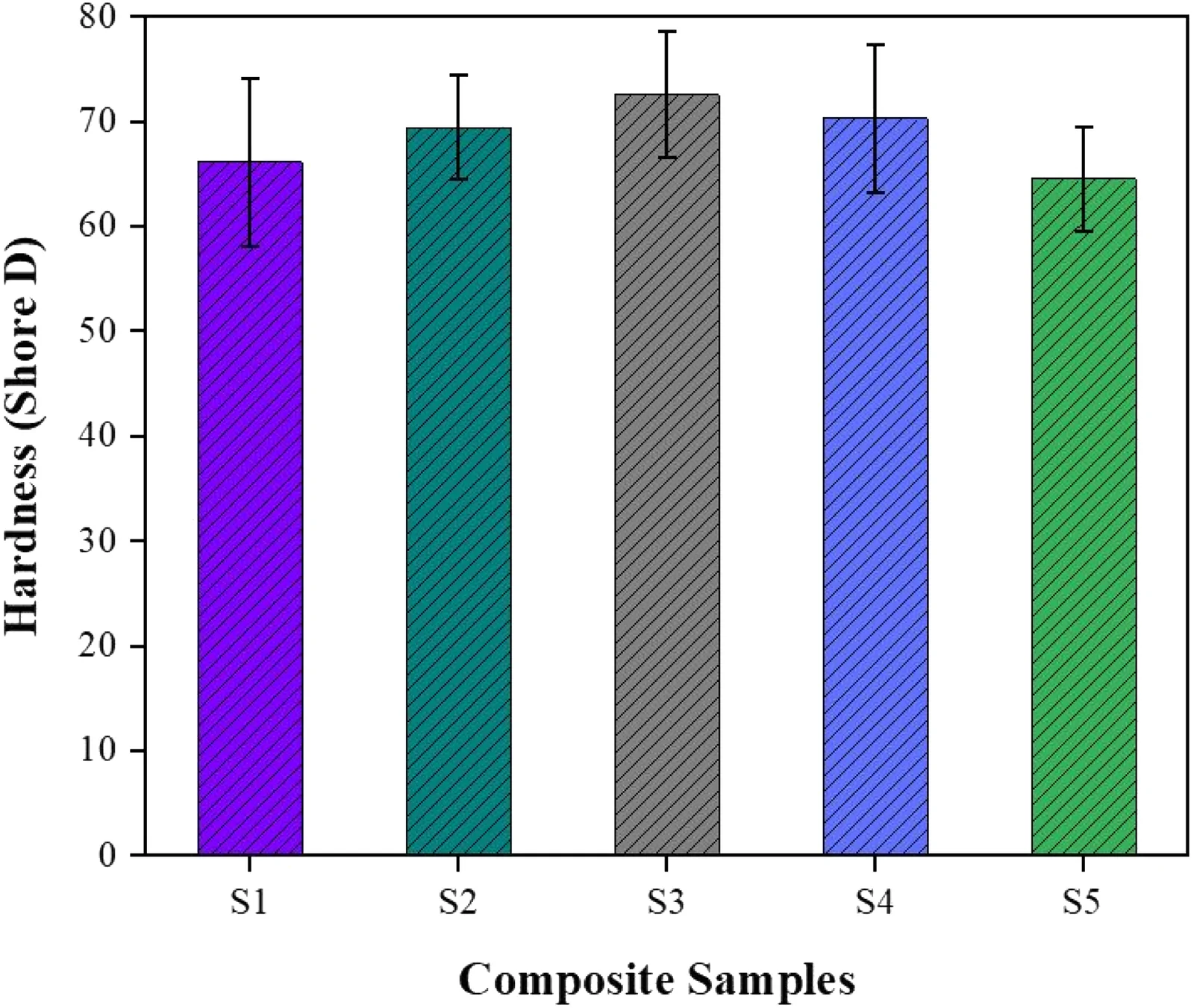
Hardness of fabricated SP/epoxy composites with varying FS bio-filler contents.

The Shore D hardness of the SP/epoxy composites reinforced with FS bio-filler in Fig. 8 demonstrates a clear dependence on filler content, showing an initial enhancement followed by a decline at higher loadings. The composite S1 exhibits a hardness of 66.12 Shore D, reflecting the effective combination of the stiff epoxy matrix and the reinforcing SP fibers, which together provide uniform surface resistance to indentation. With the introduction of 5 wt% FS bio-filler (S2), the hardness increases to 69.41 Shore D, and reaches a maximum of 72.54 Shore D at 10 wt% FS (S3). This improvement is primarily due to the rigid, mineral-rich nature of the fish scale particles, composed mainly of hydroxyapatite and collagen residues, which act as micro-reinforcements within the epoxy matrix, limiting surface deformation and enhancing load-bearing capacity.^27,38^ Beyond this optimal filler loading, however, hardness begins to decrease: 70.27 Shore D at 15 wt% FS (S4) and 64.50 Shore D at 20 wt% FS (S5). At these higher loadings, the excessive particulate content leads to poor dispersion, particle agglomeration, and reduced matrix continuity, which in turn creates microvoids and weak interfacial regions.^26,39^ These structural imperfections facilitate localized surface deformation under indentation, thereby reducing hardness.^36^ Overall, the data indicate that moderate FS incorporation strengthens the composite surface by enhancing stiffness and restricting matrix deformation, whereas excessive FS disrupts the microstructure, diminishes effective fiber–matrix interaction, and weakens resistance to indentation, demonstrating the critical balance between filler reinforcement and matrix continuity for optimal hardness performance.

### 3.5 FTIR analysis

**Fig. 9:**
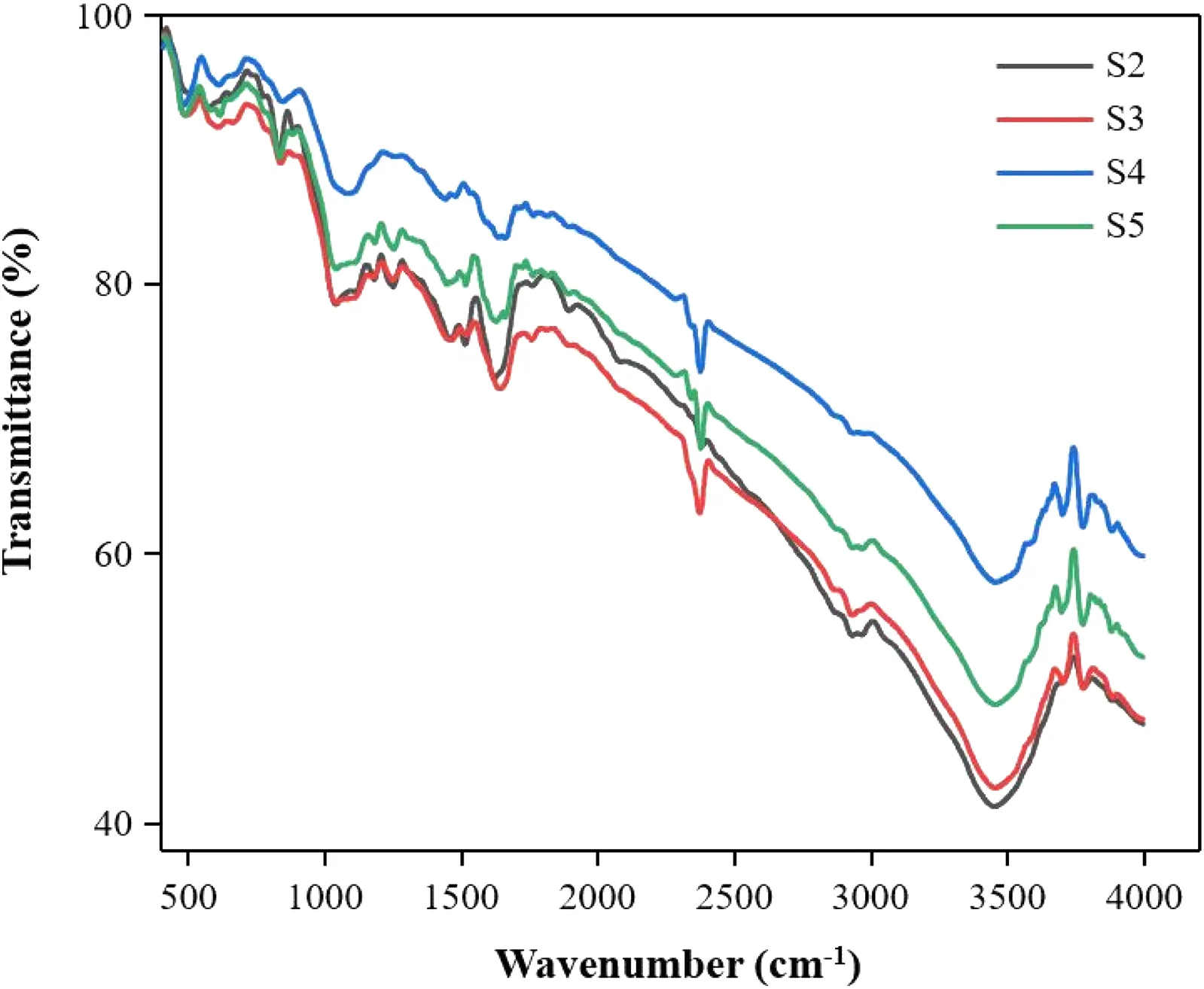
FTIR analysis of fabricated SP/epoxy composites with varying contents of FS bio-filler (5–20 wt%).

Fig. 9 displays the FTIR spectra of SP/epoxy composites reinforced with varying FS bio-filler contents (S2–S5) over the wavenumber range of 500–4000 cm^−1^. All spectra exhibit characteristic absorption bands associated with lignocellulosic fibers, epoxy matrix, and mineral-rich fish scale constituents, confirming the coexistence of these phases within the composite structure. A broad absorption band observed around 3200–3500 cm^−1^ corresponds to O–H stretching vibrations, primarily attributed to hydroxyl groups present in snake plant fibers (cellulose and hemicellulose) and residual bound moisture. The intensity and slight broadening of this band with increasing FS content (notably in S4 and S5) suggest enhanced hydrogen-bonding interactions, possibly due to the presence of collagen residues and polar functional groups within the fish-scale bio-filler.^9,40,41^

The absorption peaks near 2920–2850 cm are assigned to asymmetric and symmetric C–H stretching vibrations of aliphatic –CH_2_ and –CH_3_ groups, arising from both the epoxy matrix and lignocellulosic components of SP fibers. The distinct peak around 1720–1740 cm^−1^, though relatively weak, can be associated with C=O stretching of ester or carbonyl groups, indicative of hemicellulose content and residual curing reactions in the epoxy system. A prominent band observed near 1600–1650 cm^−1^ corresponds to aromatic C=C stretching of the epoxy resin backbone as well as possible amide I vibrations from collagen present in fish scales.^28,41^ The region between 1500 and 1000 cm^−1^ shows multiple overlapping peaks, including C–O–C stretching of epoxy groups (∼1100–1250 cm^−1^) and C–O stretching vibrations of cellulose, confirming the effective incorporation of SP fibers into the matrix. Importantly, the lower wavenumber region (approximately 500–900 cm^−1^) exhibits characteristic phosphate-related vibrations attributed to hydroxyapatite, the principal mineral component of fish scales. The presence and gradual prominence of bands in this region with increasing filler content (from S2 to S5) confirm the successful integration of FS bio-filler into the epoxy composite.^32^ No new significant peaks are observed, indicating that the composite fabrication primarily involves physical interactions and hydrogen bonding rather than the formation of new covalent chemical bonds. However, slight shifts in peak positions and variations in transmittance intensity suggest interfacial interactions between SP fibers, FS particles, and the epoxy matrix.

### 3.6 SEM analysis

**Fig. 10:**
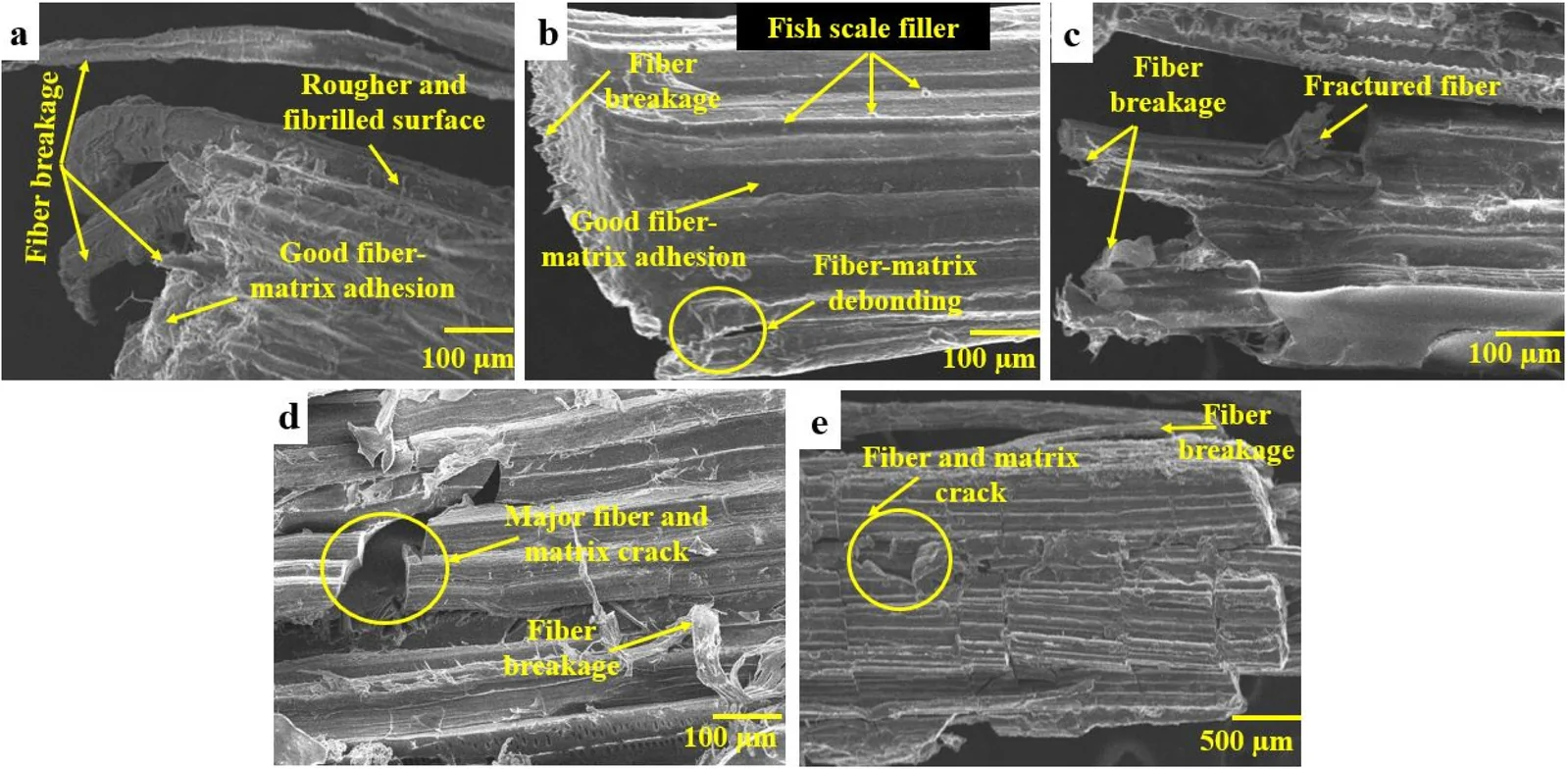
SEM micrographs of tensile-fractured specimens: (a) S1, (b) S2, (c) S3, (d) S4, and (e) S5.

Fig. 10 shows the SEM micrographs of the fractured surfaces of SP/epoxy composites with varying FS bio-filler content. The fracture morphology clearly reveals the influence of FS loading on fiber–matrix interaction, crack propagation, and failure mechanisms. In the filler-free composite S1 (a), the fractured surface exhibits a relatively rough, fibrillated fiber morphology, with evident fiber breakage and strong fiber–matrix adhesion. The presence of adhered epoxy layers on the fiber surface indicates effective interfacial bonding and efficient stress transfer between the SP fibers and the epoxy matrix. Limited voids and the absence of significant interfacial gaps indicate that the 80 wt% epoxy content ensured proper wetting and fiber encapsulation. The rough fracture surface and fibrillation imply energy absorption through fiber pull-out and fiber fracture, consistent with the superior tensile, flexural, and impact properties observed for S1.

With the introduction of 5 wt% FS bio-filler in S2 (b), the micrograph still shows relatively good fiber–matrix adhesion; however, localized fiber–matrix debonding becomes visible. The presence of fish-scale particles embedded in the matrix confirms successful filler incorporation. Although interfacial bonding remains reasonably strong, the appearance of small gaps and partial debonding regions suggests the initiation of stress concentration sites due to filler addition. In S3 (c), containing 10 wt% FS, fiber breakage becomes more substantial, and fractured fiber ends are clearly visible. The matrix appears less uniformly bonded to the fiber surface, indicating reduced interfacial strength. The increase in filler content likely disrupts matrix continuity and promotes localized stress amplification, which accelerates crack propagation under loading.

At higher filler loadings, significant microstructural deterioration is observed. In S4 (d, 15 wt% FS), major fiber and matrix cracks are evident, along with extensive fiber breakage. The circled region highlights large interfacial cracks, suggesting poor stress transfer and premature failure. The increased presence of voids and microcracks indicates that excessive filler loading reduces the effective epoxy encapsulation and promotes particle agglomeration, resulting in weak interfacial zones. In S5 (e, 20 wt% FS), severe fiber–matrix cracking and extensive fiber breakage dominate the fracture surface. The matrix appears discontinuous, and large cracks propagate along the fiber–matrix interface, indicating brittle fracture behavior. The high filler concentration likely causes agglomeration and insufficient matrix coverage, significantly weakening interfacial adhesion and facilitating rapid crack growth. The SEM analysis demonstrates a clear transition in fracture morphology from rough, well-bonded, and energy-absorbing surfaces in S1 to progressively brittle and defect-dominated structures in S4 and S5. The microstructural observations strongly correlate with the mechanical property trends, confirming that moderate filler addition introduces interfacial heterogeneities, while excessive FS loading leads to poor fiber–matrix adhesion, crack initiation, and premature composite failure.

### 3.7 Water absorption kinetics and apparent Fickian diffusion coefficient

**Fig. 11:**
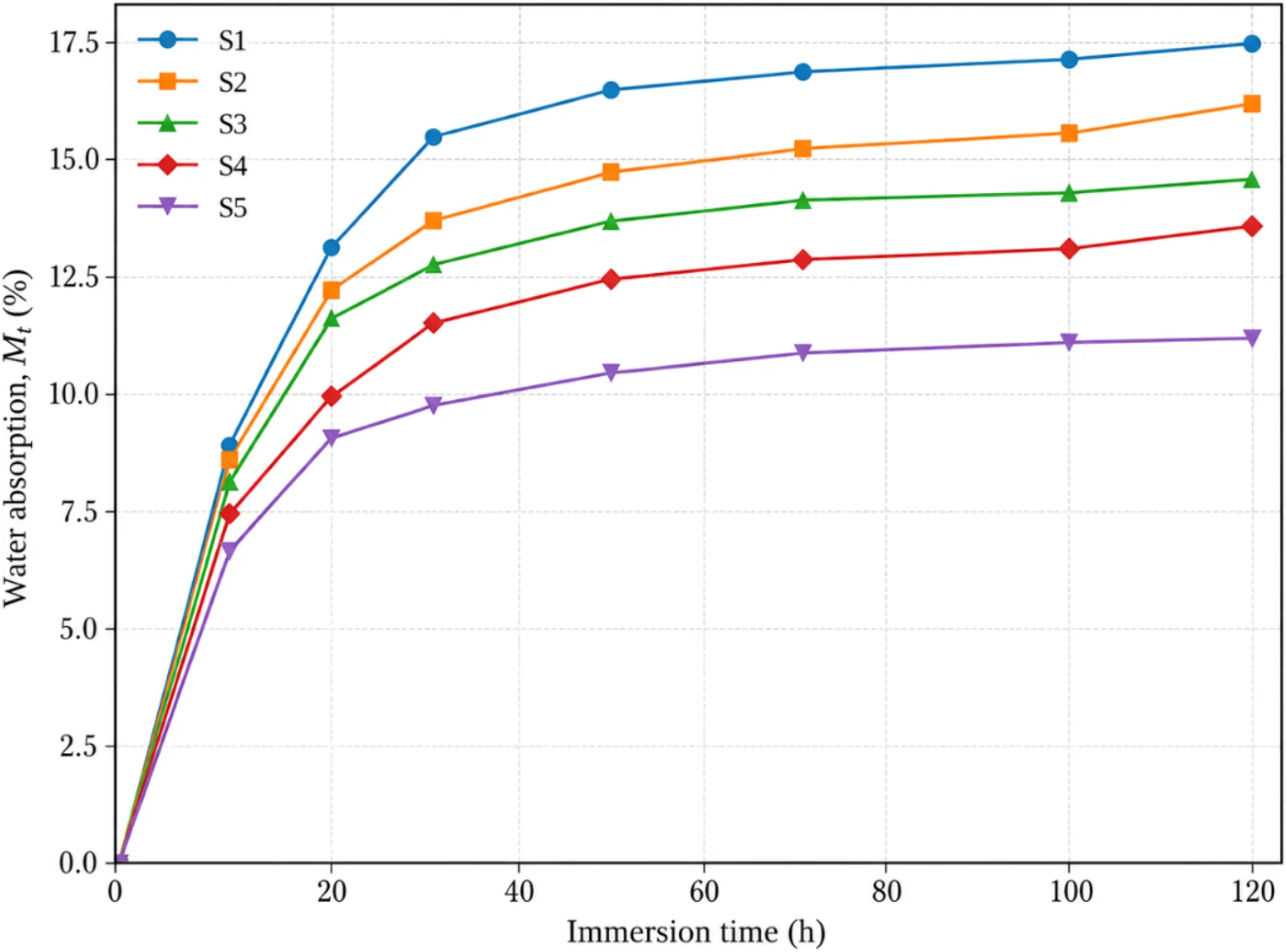
Experimental water absorption of SP/epoxy composites containing 0–20 wt% fish-scale biofiller as a function of immersion time up to 120 h.

Fig. 11 shows rapid water uptake during the first 20–30 h, followed by a progressive reduction in uptake rate as the curves approached an operational plateau. The 120 h water absorption (*M*_120_) decreased from 17.50% for S1 to 16.12%, 14.50%, 13.61%, and 11.20% for S2–S5, respectively. These values represent reductions of 7.9%, 17.1%, 22.2%, and 36% relative to S1. The initial increase is consistent with simultaneous diffusion through the epoxy-rich phase and capillary ingress along hydrophilic SP fibers, fiber–matrix interfaces, and connected defects, which are established moisture pathways in lignocellulosic polymer composites.^23,42–44^

Fish-scale powder may not be considered a purely inorganic or intrinsically hydrophobic filler because untreated scales retain a collagen-rich organic phase together with calcium-phosphate minerals.^40,41^ The progressive reduction in *M*_120_ is therefore more reasonably associated with several simultaneous effects. Substitution of epoxy with the mineral-rich particulate phase alters the number and distribution of accessible sorption sites per unit dry mass, while dispersed particles may increase tortuosity in some regions, though this effect may compete with possible interface-related pathways. Because water absorption is normalized by the initial dry mass, incorporation of a denser, mineral-rich phase may also reduce the measured percentage without eliminating local ingress pathways.^45^ The present trend agrees with that of Behera *et al.*,^46^ and they found lower water absorption in particle-filled epoxy composites containing fish-scale waste. In contrast, Rahman *et al.*^47^ reported an increase in water absorption from 0.28% to 2.52% when untreated full-form scales were incorporated into epoxy, which they related to hydrophilic collagen, weak scale–matrix interfaces, and connected voids. The contrasting results indicate that fish-scale loading alone does not determine moisture response. Scale form, organic-to-mineral composition, particle distribution, matrix substitution or addition, interfacial adhesion, and defect connectivity are also determining factors.

**Fig. 12:**
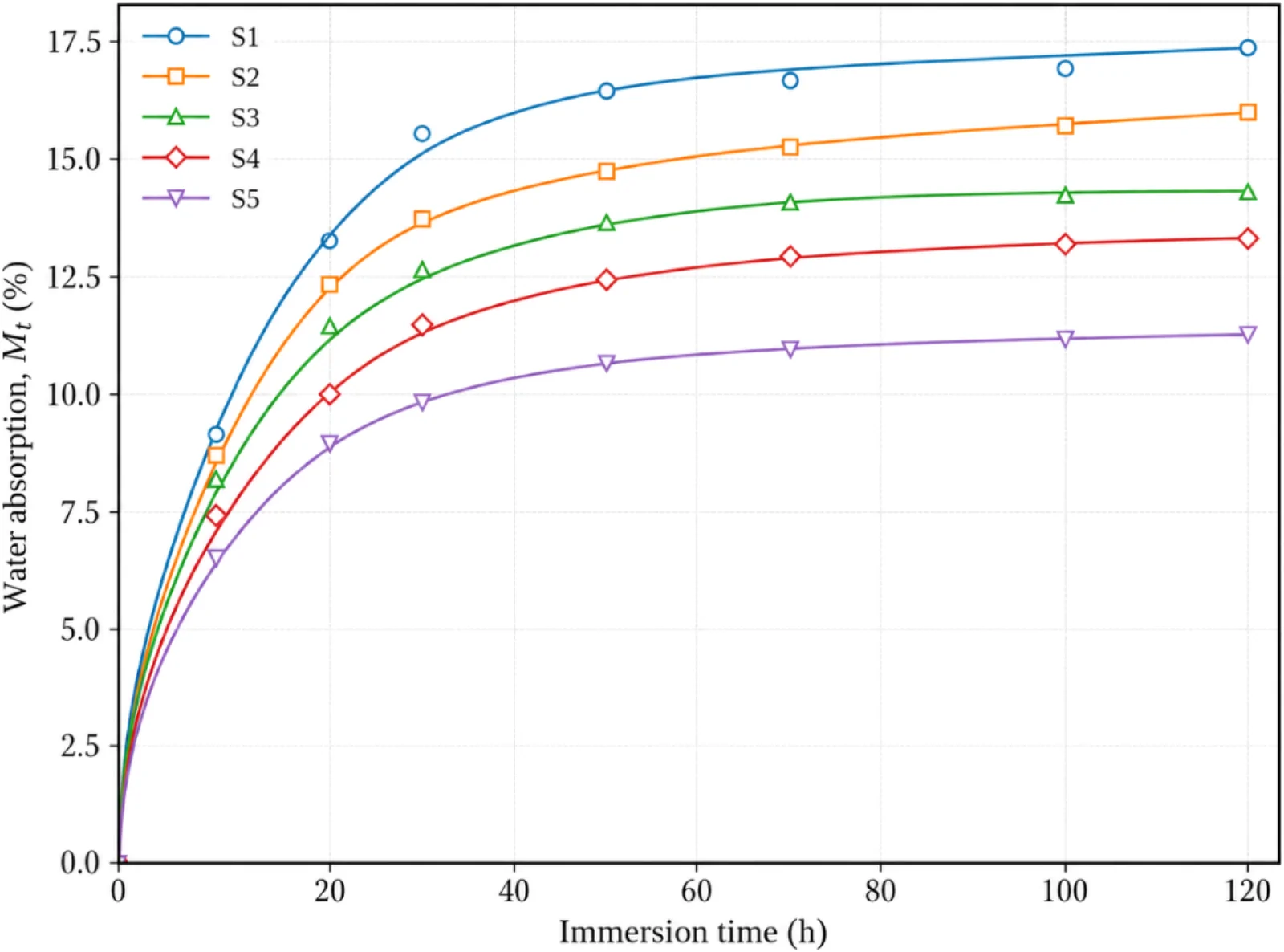
Experimental water absorption profiles and apparent Fickian model fits for SP/epoxy composites containing 0–20 wt% fish-scale biofiller. Symbols represent experimental data, while solid lines represent nonlinear least-squares fits using eqn (2), with *M*_120_ adopted as the operational plateau value.

Fig. 12 shows close agreement between the experimental values and the plane-sheet model fits over the tested interval. The model yielded *R*^2^ values of 0.993–0.998 and RMSE values of 0.202–0.411 percentage points (Table 2). However, *D*_app_ remained within 1.401–1.724 × 10^−11^ m^2^ s^−1^ and showed no consistent dependence on FS content. Notably, S5 exhibited the lowest *M*_120_ value of 11.20% but the highest *D*_app_ value of 1.724 × 10^−11^ m^2^ s^−1^. This behavior is not contradictory because gravimetric moisture retention and apparent moisture-transport rate represent distinct aspects of sorption.^23,45^ Although S5 retained less water relative to its initial dry mass, its normalized uptake curve approached the operational plateau comparatively rapidly. However, the higher-fitted *D*_app_ of S5 should not be considered as evidence of faster bulk diffusion alone, because the fitted parameter reflects the combined effects of molecular diffusion, interface-controlled transport, and defect-assisted moisture pathways.

**Table 2:** Apparent Fickian diffusion parameters of SP/epoxy composites containing different fish-scale biofiller contents (0–20 wt%)^a^

Sample	FS (wt%)	*M*_120_ (%)	Reduction *vs.* S1 (%)	*D*_app_ (×10^−11^ m^2^ s^−1^)	*R* ^2^	RMSE (percentage points)
S1	0	17.50	0	1.454	0.996	0.357
S2	5	16.12	7.9	1.423	0.993	0.411
S3	10	14.50	17.1	1.656	0.998	0.228
S4	15	13.61	22.2	1.401	0.995	0.294
S5	20	11.20	36.0	1.724	0.997	0.202

a *M*_120_ was used as an operational plateau value for comparative fitting. *R*^2^ and RMSE indicate the quality of the fit and do not reflect between-specimen variability.

The tensile-fracture micrograph of S5 (Section 3.6) reveals extensive cracking, fiber–matrix separation, discontinuous matrix regions, and void defects, indicating interfacial discontinuities that may promote defect-assisted moisture ingress. Moreover, FTIR analysis showed no evidence of new covalent interactions or chemically formed moisture barriers. Therefore, the lower gravimetric water uptake of S5 does not necessarily indicate effective microvoid filling or sealing; rather, reduced moisture retention may coexist with faster transport through interconnected interfacial pathways.

The model-fit results should be considered within the methodological limitations described in Section 2.10 and do not establish specimen-to-specimen repeatability, parameter identifiability, or long-term predictive validity. Within these constraints, FS substitution reduced the 120 h gravimetric water uptake by up to 36% but did not systematically decrease the apparent moisture-transport rate. Longer-duration immersion, replicate-based uncertainty quantification, dimensional-swelling measurements, and wet-state mechanical-retention testing are therefore needed before improved long-term moisture durability can be conclusively established.

## 4 Conclusions

This study systematically investigated the effect of FS bio-filler incorporation on the mechanical behavior, microstructural characteristics, and moisture resistance of SP fiber-reinforced epoxy composites. Based on the experimental results, the major findings are summarized as follows:

• The filler-free composite exhibited the maximum tensile, flexural, and impact properties, demonstrating effective stress transfer due to good fiber–matrix interaction and matrix continuity.

• The incorporation of FS bio-filler progressively reduced the tensile and impact properties, while the flexural properties showed a non-monotonic trend, with a slight improvement at 10 wt% FS followed by deterioration at higher filler loadings.

• Hardness peaked at 10 wt% FS, while *M*_120_ decreased by 36%, from 17.50% in S1 to 11.20% in S5. However, apparent diffusion coefficients varied between 1.401 and 1.724 × 10^−11^ m^2^ s^−1^ without a consistent trend, indicating reduced moisture retention but not consistently slower transport.

• SEM observations revealed increased defects, localized agglomeration, and filler pull-out at higher FS contents, while FTIR confirmed physical interactions among the composite constituents without the formation of new chemical bonds.

• Overall, 5–10 wt% FS offered the best performance balance, whereas 20 wt% maximized moisture reduction but caused the greatest tensile and impact losses.

The fabricated composites are lightweight, sustainable, and bio-based and may be considered for non-load-bearing and selected semi-structural applications, including interior automotive components, wall and ceiling panels, furniture components, and protective casings, where mechanical requirements are moderate. However, the deterioration in mechanical properties at higher FS loadings restricts their use in primary load-bearing structures, and the present 120 h immersion results alone do not establish long-term moisture durability. Future work may combine environmentally friendly fiber/filler surface treatments and improved dispersion with longer-duration immersion, replicate-based uncertainty quantification, dimensional-swelling measurements, wet-state mechanical-retention testing, and edge-sealed directional diffusion experiments. Advanced chemical characterization, including quantitative surface-functional-group analysis, curing kinetics, and molecular-level interfacial investigation, can further clarify interactions among the epoxy matrix, SP fibers, and FS bio-fillers.

## Author contributions

Barshan Dev: conceptualization, methodology, investigation, supervision, writing – original draft; Md Ashikur Rahman: methodology, investigation, visualization, writing – original draft; Apurbo Das: investigation, formal analysis; Md Azmol Hossain Emon: investigation, formal analysis; Sakib Iqbal Akand: resources, data curation; Abhi Karmakar: resources, data curation; Md Zillur Rahman: methodology, validation, supervision, writing – original draft, writing – review and editing.

## Conflicts of interest

The authors declare that there are no conflicts of interest related to this work.

## Data Availability

All relevant data and materials can be provided by the authors upon reasonable request.
